# A multi-center study on the association between serum magnesium levels and allostatic load in hemodialysis patients

**DOI:** 10.3389/fphys.2022.963914

**Published:** 2022-10-03

**Authors:** Yingxin Zhang, Zhengling Yang, Huan Yang, Xiuyong Li, Zhi Liu, Youwei Bai, Guangrong Qian, Han Wu, Ji Li, Yuwen Guo, Shanfei Yang, Lei Chen, Jian Yang, Jiuhuai Han, Shengyin Ma, Jing Yang, Linfei Yu, Runzhi Shui, Xiping Jin, Hongyu Wang, Fan Zhang, Tianhao Chen, Xinke Li, Xiaoying Zong, Li Liu, Jihui Fan, Wei Wang, Yong Zhang, Guangcai Shi, Deguang Wang, Shuman Tao

**Affiliations:** ^1^ Department of Nephrology, The Second Hospital of Anhui Medical University, Hefei, China; ^2^ Blood Purification Center, No. 2 People’s Hospital of Fuyang City, Fuyang, China; ^3^ Department of Nephrology, The First Affiliated Hospital of Anhui University of Science and Technology, Huainan, China; ^4^ Department of Nephrology, The Second People’s Hospital of Lu’an City, Lu’an, China; ^5^ Department of Nephrology, Maanshan People’s Hospital, Maanshan, China; ^6^ Blood Purification Center, Bozhou People’s Hospital, Bozhou, China; ^7^ Department of Nephrology, Tongling People’s Hospital, Tongling, China; ^8^ Department of Nephrology, Lujiang County People’s Hospital, Lujiang, China; ^9^ Department of Nephrology, Shouxian County Hospital, Shouxian County, China; ^10^ Department of Nephrology, Hefei Jinnan Kidney Hospital, Hefei, China; ^11^ Department of Nephrology, Funan County People’s Hospital, Funan County, China; ^12^ Department of Nephrology, Anqing Municipal Hospital, Anqing, China; ^13^ Department of Nephrology, Anhui Wanbei Coal-Electricity Group General Hospital, Suzhou, China; ^14^ Department of Nephrology, The First People’s Hospital of Hefei, Hefei, China; ^15^ Department of Nephrology, The People’s Hospital of Taihu, Taihu County, China; ^16^ Blood Purification Center, Huangshan City People’s Hospital, Huangshan, China; ^17^ Department of Nephrology, Huainan Chao Yang Hospital, Huainan, China; ^18^ Department of Nephrology, Lixin County People’s Hospital, Lixin County, China; ^19^ Department of Nephrology, Dongzhi County People’s Hospital, Dongzhi County, China; ^20^ Department of Nephrology, Tianchang City People’s Hospital, Tianchang, China; ^21^ Department of Nephrology, Xiaoxian People’s Hospital, Xiaoxian County, China; ^22^ Department of Nephrology, The Second Affiliated Hospital of Bengbu Medical College, Bengbu, China; ^23^ Department of Nephrology, The Second People’s Hospital of Hefei, Hefei, China; ^24^ Department of Nephrology, Huaibei People’s Hospital, Huaibei, China; ^25^ Department of Nephrology, The People’s Hospital of Xuancheng City, Xuancheng, China; ^26^ Department of Nephrology, Lujiang County Hospital of TCM, Lujiang County, China; ^27^ Department of Nephrology, The Fifth People’s Hospital of Hefei, Hefei, China

**Keywords:** allostatic load, magnesium, hemodialysis, clinical, stress

## Abstract

**Objective:** Serum magnesium (Mg^2+^) levels are associated with insulin resistance, hypertension, lipid abnormalities, and inflammation. However, limited studies have indicated the relationship between Mg^2+^ and multiple system indexes. The purpose of this study was to investigate the association between Mg^2+^ and allostatic load (AL) in hemodialysis patients.

**Methods:** A cross-sectional survey was conducted on hemodialysis patients from different centers in Anhui Province, China, between January and December 2020. A total of 3,025 hemodialysis patients were recruited. Their clinical data were measured before hemodialysis. Information was collected by an online self-reported questionnaire and medical record. Serum Mg^2+^ was divided into three groups by tertiles. A score of AL greater than or equal to 3 was defined as high AL. A binary logistic regression model was applied to examine the relationship between serum Mg^2+^ and AL.

**Results:** A total of 1,222 patients undergoing hemodialysis were included, 60% of whom were males (733/1,222). The mean (standard deviation) age of patients was 55.90 (12.75). The median level of serum Mg^2+^ was 1.22 mmol/L. The rate of high AL levels was 23.4%. Serum Mg^2+^ was negatively correlated with body mass index, fasting blood glucose (Glu), and C-reactive protein and positively correlated with high-density lipoprotein, low-density lipoprotein, total cholesterol, diastolic blood pressure (DBP), and serum phosphorus. After adjusting for gender, anxiety, diabetes, family residence, lipid-lowering agents, antihypertensive medications, albumin, and Glu, the binary logistic regression model showed that patients with lower levels of serum Mg^2+^ were more likely have high AL (*OR* for the T1 group of serum Mg^2+^:1.945, 95% *CI*: 1.365–2.773, and *OR* for the T2 group of serum Mg^2+^:1.556, 95% *CI*: 1.099–2.201).

**Conclusion:** Our data support the hypothesis that higher serum Mg^2+^ concentrations may contribute to lower health risk in hemodialysis populations. Further randomized controlled trials and cohort studies are warranted to verify whether Mg^2+^ supplementation could be part of routine examinations in hemodialysis populations.

## Introduction

End-stage renal disease (ESRD) represents the final phase of kidney deterioration and is characterized by an estimated GFR of <15 ml/min/1.73 m^2^, with the occurrence of signs or symptoms of uremia (Carlo et al., 2015). Hemodialysis is the main alternative therapy for sustaining life in ESRD patients (Ahmadmehrabi and Tang, 2018). Every year, about 1 million patients with ESRD are undergoing hemodialysis around the world. The number of hemodialysis patients has been increasing at a global average annual rate of 7% (Shinkawa et al., 2019). More than 1.5 million ESRD patients were reported in China, and 100,000 to 150,000 new cases are reported each year (Xiao et al., 2011). Poor prognosis and high medical costs for hemodialysis patients have become a global public health problem, which seriously puts a heavy burden on health conditions and economic status. In addition, hemodialysis often aggravates physiological and psychological problems, such as calcium and phosphorus metabolism disorders, bone abnormalities, and psychological stress. Some studies showed that stress played an important role in health among hemodialysis patients (García-Llana et al., 2014). However, few studies have comprehensively assessed stress in the hemodialysis population by objective measures.

The concept of allostatic load (AL) was proposed by McEwen and Stellar (1993). AL is defined as the cumulative burden of chronic stress and life events (Guidi et al., 2021). It is also a comprehensive index that reflects multiple biological system disorders and can be used as an early predictor of health risks (Guidi et al., 2021; Nelson et al., 2021). The algorithms of a multisystemic AL index have been developed in some epidemiological studies, which was the sum of various clinical indications of multiple physiological systems that reached subclinical thresholds (Seeman et al., 1997). In recent studies, AL is commonly assessed by three biological systems, namely, metabolic system, autonomic nervous system, and immune system. A higher level of AL is commonly connected to chronic disease (Sabbah et al., 2008; Duru et al., 2012) and it has also been proven that it had a negative impact on chronic kidney disease (Bruce et al., 2015; Norton et al., 2016). In addition, some clinical indicators may also affect AL.

Magnesium (Mg^2+^) is the fourth most abundant cation in the human body, which is found primarily in bone and skeletal muscle (Navarro-González et al., 2009). The importance of Mg^2+^ on health has been emphasized by Kruse et al. (1934). Clinical and experimental evidence suggested that serum Mg^2+^ concentrations declined in humans or animals with chronic disorders (Romani, 2011). Hypomagnesemia was a common complication in dialysis patients with ESRD. Serum Mg^2+^ levels exceeding 0.9 mmol/L were found in 31.5% of hospitalized patients, whereas levels below 0.7 mmol/L were found in 20.2% of patients (Cheungpasitporn et al., 2015). Dietary limitations, use of loop and thiazide diuretics, and a reduced Mg^2+^ concentration in the dialyzate all contribute to Mg^2+^ loss (Navarro-González et al., 2009). Mg^2+^ may play a vital role in the literature linking magnesium derangements with bone disease, cardiovascular disease, sudden cardiac death, and mortality (Misra and Nessim, 2017; Floege, 2018). A low serum Mg^2+^ level was thought to contribute to endothelial dysfunction, soft tissue calcification, and cardiac arrhythmias (Shechter et al., 2000; Kanbay et al., 2012). However, the effects of magnesium homeostasis disturbances on multiple biological system disorders in hemodialysis patients are still not well-recognized.

Some studies have attempted to confirm the effects of different Mg^2+^ levels on health and its potential pathway among patients with chronic diseases. However, the associations between serum Mg^2+^ and AL are not well-established, particularly in patients undergoing hemodialysis. Therefore, we hypothesize that patients undergoing hemodialysis with low levels of serum Mg^2+^ may have a greater risk of high-level AL. The present study was conducted with a sample size of 1,222 patients in 27 blood purification centers, which could be beneficial in providing evidence for the clinical effectiveness of magnesium supplementation.

## Materials and methods

### Participants

A multi-center, cross-sectional study was conducted among patients undergoing hemodialysis from 27 blood purification centers of Anhui Province located in East China between 1 January and 31 December 2020. A total of 3,025 patients were recruited. Inclusion criteria for participants were age ≥18 years old with regular hemodialysis for more than 3 months and provided informed consent. Patients who were <18 years old or refused to participate or were defined as follows: heart failure with NYHA Ⅲ grade or above; complicated with severe liver, lung, brain, and other organ failure diseases, such as cirrhosis, chronic respiratory failure, and hemiplegia; HIV infection or AIDS; complications with malignant tumor psychosis were excluded. A total of 1,803 patients were excluded without C-reactive protein (CRP) data, which was a biological marker of AL. Finally, 1,222 patients were analyzed in the study.

This study was approved by the Ethics Committee of the Second Hospital of Anhui Medical University (No. PJ-YX2020-006). Electronic informed consent was obtained from all participants before completing the survey. The study was conducted in accordance with the principles of the Declaration of Helsinki.

### Basic information

An online platform named “h6world” supported by the Clinical Big Data Platform Research Group of Peking University was used to collect data. Before the survey, doctors, nurses, or graduate students of each center were trained. Each center logged into the platform for investigation data entry. All data were summarized to the project leader and were checked by a quality controller, including the demographic characteristics (e.g., gender, age, family residence, family income, etc.), behaviors (e.g., smoking, drinking, etc.), hemodialysis status, psychological states, medications, comorbidities, and clinical data.

### Physical examination

For all patients, anthropometric parameters such as body mass index (BMI), systolic blood pressure (SBP), diastolic blood pressure (DBP), and blood samples were collected before hemodialysis. Height measurement is accurate to 0.01 m, and weight measurement is accurate to 0.1 kg, measured with a calibrated electronic scale. The BMI was calculated as weight (kg) divided by height squared (square meters) (kg/m^2^).

### Allostatic load

Laboratory analyses were performed on fasting venous blood samples collected before dialysis. AL levels in the hemodialysis population were comprehensively assessed using three biological systems: the metabolic system which contains total cholesterol (TC), high-density lipoprotein (HDL), low-density lipoprotein (LDL), triglyceride (TG), and BMI; autonomic nervous system (SBP and DBP); immune system containing CRP and white blood cells (WBCs). The cut-off points for blood pressure and BMI were based on the KDOQI Clinical Practice Guidelines for Cardiovascular Disease in Dialysis Patients (K/DOQI Workgroup, 2005) and WHO Expert Consultation about the Asian criteria of obesity (WHO Expert Consultation, 2004). The cut-off points for blood lipids in the metabolic system were determined according to the 2016 Chinese guideline for the management of dyslipidemia in adults (Joint committee issued Chinese guideline for the management of dyslipidemia in adults, 2016). The normal range for WBC count was 4–10 × 10^6^/L and for CRP was 0–10 mg/L, which was determined based on clinical laboratory criteria (Davarpanah and Eberhardt, 2020). These data were measured by standard methods in the department of biochemistry. The specific threshold standards are listed in Table 1.

**TABLE 1:** Threshold standard for biomarkers of allostatic load.

Biomarker	M ± SD	Threshold standard		
		Cut-off point	*n* (%)	Scoring
Metabolic system				
HDL-C (mmol/L)	1.11 ± 0.52	<1.0	462 (37.8)	1
LDL-C (mmol/L)	2.24 ± 1.90	≥4.1	17 (1.4)	1
TG (mmol/L)	1.83 ± 1.95	≥2.3	210 (17.2)	1
TC (mmol/L)	4.33 ± 5.43	≥6.2	23 (1.9)	1
BMI (kg/m^2^)	21.70 ± 3.36	≥25	182 (14.9)	1
Autonomic nervous system				
SBP (mm Hg)	143.24 ± 19.49	≥140	777 (63.6)	1
DBP (mm Hg)	82.02 ± 29.20	≥90		
Immune system				
WBC (10^9^/L)	6.27 ± 3.20	4–10	185 (15.1)	1
CRP (mg/L)	9.45 ± 28.11	>10	224 (18.3)	1
Allostatic load	1.70 ± 1.12	≥3	286 (23.4)	—

Note: TG , triglyceride; TC, total cholesterol; HDL, high-density lipoprotein; LDL, low-density lipoprotein; BMI, body mass index; SBP, systolic blood pressure; DBP, diastolic blood pressure; WBC, white blood cell; CRP, c-reactive protein.

Each biomarker was assigned a value according to the threshold value standard, and the value exceeding the threshold value (HDL was below the threshold value) is assigned a value of 1, otherwise, it is assigned a value of 0. The total scores of AL were obtained by adding up all the indexes, ranging from 0 to 8. High scores mean that patients were suffering a more serious imbalance of the biological system. The 75th percentile of the AL scores was 2. Therefore, participants with total scores ≥3 are defined as the high-AL group, as found in previous studies (Peek et al., 2010; Frei et al., 2015).

### Statistical analysis

The measurement data were represented by the mean and standard deviation (SD) for normally distributed data and by median and quartile for skewed distribution data. Two independent *t*-tests and rank sum tests were used to examine the difference in clinical information between high-AL and low-AL groups. Categorical data were expressed as frequencies (n) and percentages (%). The chi-square test was used to calculate the difference in the percentage of high AL between different groups. Spearman’s correlations were applied to analyze the correlations between serum Mg^2+^ and clinical index in patients undergoing hemodialysis. AL scores were counted by the cut-off points of eight biomarkers and calculated by numerical transformations. It was not a distribution of probability and was presented as a dichotomous variable. Therefore, binary logistic regression was used to analyze the association between serum Mg^2+^ and AL. These data were processed by SPSS 26.0. All statistical tests were two-sided, and the significance was set at *p* < 0.05.

## Results

### Characteristics of patients undergoing hemodialysis

Table 2 lists the demographic and clinical characteristics of 1,222 patients recruited in this study, including 733 males (60.0%) and 489 females (40.0%). The mean age of hemodialysis patients was 55.9 (SD: 12.75), and the percentage of the high level of AL was 23.4% (286/1,222). The median (Q1 and Q3) hemodialysis vintage was 4.35 (2.08 and 7.08) years. The proportion of high AL level was higher in males (26.3%) and lower in females (19.0%). The proportion of high AL level was higher in urban population (26.8%) than in rural population (18.4%). In addition, patients without anxiety disorder reported a higher rate of high AL levels (26.5%). Patients with diabetes reported a higher rate of high AL levels (31.0%). Patients with diabetes reported a higher rate of high AL level (31.0%) than patients without diabetes (20.5%). Patients taking antihypertensive medications reported a lower rate of high AL levels (20.4%) than patients not taking antihypertensive medications (29.1%). However, patients taking hypolipidemic medications reported a higher rate of high AL levels (34.8%) than patients not taking hypolipidemic medications (20.3%). In addition, a lower level of Alb and higher level of Glu were found in the high-AL group. All the aforementioned indexes had statistical significance (*p* < 0.05).

**TABLE 2:** Characteristics of patients undergoing hemodialysis (*n* = 1,222).

Variables	Total [M ± SD/*n* (%)/Median (Q1, Q3)]	Allostatic load		*P* value
		<3 (*n* = 936)	≥3 (*n* = 286)	
Age (years)	55.90 ± 12.75	55.70 ± 12.70	56.56 ± 12.90	0.323
Gender				0.004
Males	733 (60.0)	540 (73.7)	193 (26.3)	
Females	489 (40.0)	396 (81.0)	93 (19.0)	
Family residence				0.001
Urban	728 (59.6)	533 (73.2)	195 (26.8)	
Rural	494 (40.4)	403 (81.6)	91 (18.4)	
Family income				0.901
≤¥4,000	366 (30.0)	279 (76.2)	87 (23.8)	
>¥4,000	856 (70.0)	657 (76.8)	199 (23.2)	
Smoking				0.304
Yes	184 (15.1)	135 (73.4)	49 (26.6)	
No	1,038 (84.9)	801 (77.2)	237 (22.8)	
Drinking				0.553
Yes	148 (12.1)	110 (74.3)	38 (25.7)	
No	1,074 (87.9)	826 (76.9)	248 (23.1)	
Anxiety				0.005
Yes	311 (25.5)	257 (82.6)	54 (17.4)	
No	911 (74.5)	679 (74.5)	232 (25.5)	
Comorbidities				
Diabetes	336 (27.5)	232 (69.0)	104 (31.0)	<0.001
Hypertension	989 (80.9)	751 (75.9)	238 (24.1)	0.300
Cardiovascular disease	194 (15.9)	140 (72.2)	54 (27.8)	0.134
Medications				
Antihypertensive	800 (65.5)	637 (79.6)	163 (20.4)	0.001
Hypolipidemic	264 (21.6)	172 (65.2)	92 (34.8)	<0.001
Apply iron				0.121
Yes	499 (40.8)	394 (79.0)	105 (21.0)	
No	723 (59.2)	542 (75.0)	181 (25.0)	
Ca (mmol/L)	2.29 (2.13, 2.43)	2.29 (2.14, 2.44)	2.29 (2.12, 2.42)	0.457
P (mmol/L)	1.82 (1.47, 2,24)	1.82 (1.47, 2.23)	1.84 (1.45, 2.26)	0.764
iPTH (pg/ml)	282.60 (127.35, 477.10)	276.15 (126.18, 470.25)	307.75 (133.30, 531.45)	0.247
Alb (g/L)	42.70 (39.00, 46.20)	43.00 (39.10, 46.50)	42.00 (38.15, 45.00)	0.001
Glu (mmol/L)	5.30 (4.60, 6.62)	5.20 (4.56, 6.62)	5.60 (4.76, 8.10)	<0.001
Hb (g/L)	110.0 (95.00, 123.00)	110.00 (96.00, 123.00)	108.50 (91.75, 122.25)	0.272
Dialysis vintage	4.35 (2.08, 7.08)	4.00 (1.99, 7.22)	4.54 (2.44, 7.00)	0.343

Note: Alb, albumin; Hb, hemoglobin; Ca, serum calcium; P, Serum phosphorus; iPTH, immunoreactive parathyroid hormone; Glu, glucose.

### Spearman’s correlations between serum Mg^2+^ and clinical data

Table 3 shows the results of Spearman’s correlations between serum Mg^2+^ and clinical indexes in patients undergoing hemodialysis. Serum Mg^2+^ was positively correlated with HDL (*r* = 0.212, *p <* 0.001), LDL (*r* = 0.230, *p <* 0.001), TC (*r* = 0.230, *p <* 0.001), DBP (*r* = 0.157, *p <* 0.001), and P (*r* = 0.176, *p <* 0.001) and negatively correlated with Glu (*r* = −0.116, *p <* 0.001), BMI (*r* = −0.085, *p = 0.003*), and CRP (*r* = −0.058, *p =* 0.043). All the aforementioned indexes had statistical significance (*p <* 0.05). There was no correlation between serum Mg^2+^ and TG, Ca, iPTH, WBC, or SBP.

**TABLE 3:** Spearman’s correlations between serum Mg^2+^ and clinical data.

Variable	Correlation coefficient	P-value
HDL-C (mmol/L)	0.212	<0.001
LDL-C (mmol/L)	0.230	<0.001
TG (mmol/L)	0.017	0.557
TC (mmol/L)	0.230	<0.001
BMI (kg/m^2^)	−0.085	0.003
SBP (mm Hg)	0.048	0.093
DBP (mm Hg)	0.157	<0.001
WBC (×10^9^/L)	0.004	0.879
CRP (mg/L)	−0.058	0.043
Alb (g/L)	0.149	<0.001
Glu (mmol/L)	−0.116	<0.001
Hb (g/L)	0.085	0.003
Ca (mmol/L)	0.001	0.968
P (mmol/L)	0.176	<0.001
iPTH (pg/ml)	0.021	0.469

Note: TG, triglyceride; TC, total cholesterol; HDL, high-density lipoprotein; LDL, low-density lipoprotein; BMI, body mass index; SBP, systolic blood pressure; DBP, diastolic blood pressure; WBC, white blood cell; CRP, c-reactive protein; Alb, albumin; Hb, hemoglobin; Ca, serum calcium; P, Serum phosphorus; iPTH, immunoreactive parathyroid hormone; Glu, glucose.

### The percentage of allostatic load levels in different groups of serum Mg^2+^ among patients undergoing hemodialysis

As seen in Table 4, serum Mg^2+^ was divided into three groups by tertiles. The T1 group was defined as having serum Mg^2+^ concentration <1.00 mmol/L, the T2 group was defined as having serum Mg^2+^ concentration of 1.00–1.28 mmol/L, and the T3 group for serum Mg^2+^ concentration ≥1.28 mmol/L. The proportion of high AL levels in the T1 group (34.0%) was higher than that of the T2 (25.4%) and T3 (16.6%) groups, and the difference was statistically significant (*p <* 0.001).

**TABLE 4:** Percentage of AL levels in different groups of serum Mg^2+^ among patients undergoing hemodialysis.

Serum Mg^2+^	Total [*n* (%)]	Allostatic load [*n* (%)]		*χ* ^2^ value	*P-*value
		<3	≥3		
T1 group	297 (24.3)	196 (66.0)	101 (34.0)	34.171	<0.001
T2 group	358 (29.3)	267 (74.6)	91 (25.4)		
T3 group	567 (46.4)	474 (83.4)	94 (16.6)		

### The association between serum Mg^2+^ and allostatic load among patients undergoing hemodialysis

Table 5 depicts the crude and adjusted OR (95% CI) for serum Mg^2+^ in comparison with that of the reference group (T3 group) for AL. In model 1, the univariate logistic regression results showed that compared with the T3 group, the *OR*s for the T1 and T2 groups were 2.593 (95% CI: 1.871–3.594) and *1.715* (95% *CI*: 1.239–2.373), respectively. In model 2, after adjusting for gender, anxiety, diabetes, family residence, antihypertensive and hypolipidemic medications, and Alb and Glu, the results showed that compared with the T3 group, the *OR*s for the T1 and T2 groups were 1.945 (95% CI: 1.365–2.773) and 1.556 (95% CI: 1.099–2.201), respectively.

**TABLE 5:** Association between serum Mg^2+^ and AL among patients undergoing hemodialysis.

Serum Mg^2+^	Model 1		*OR*	95% *CI*	Model 2		*OR*	95% *CI*
	*B-*value	*P-*value			*B-*value	*P-*value		
T1 group	0.953	<0.001	2.593	1.871–3.594	0.665	<0.001	1.945	1.365–2.773
T2 group	0.539	0.001	1.715	1.239–2.373	0.442	0.013	1.556	1.099–2.201
T3 group	—	—	1.000	—	—	—	1.000	—

Note: Adjusting for gender, anxiety, diabetes, family residence, Alb, Glu, and antihypertensive and hypolipidemic medications.

### Sensitivity analysis of characteristics between the included sample and excluded sample

A sensitivity analysis was performed on the excluded samples and included samples. As shown in Supplementary Table S1, older and more urban patients were included in the analyzed samples. Additionally, the included sample showed greater rates of diabetes (27.5%), hypertension (80.9%), cardiovascular disease (15.9%), and hypolipidemic drugs (21.6%) but a lower rate of antihypertensive medications (73.0%).

## Discussion

This is the first study to analyze the correlation between serum Mg^2+^ and AL. The results showed that a high AL level was common in patients undergoing hemodialysis and was inversely related to serum Mg^2+^ concentration. The proportion of patients with high levels of AL was 23.4% in our study. Compared with other studies, patients undergoing hemodialysis had almost the same proportion in high levels of AL. For example, a recent pilot study found that the percentage of higher AL levels was 25% among female outpatients with fibromyalgia (Leombruni et al., 2019). However, the percentage of high AL levels may vary in different standards of AL. The percentage of high AL in adolescents was 35% according to the cut-off point of 2 (Theall et al., 2012). AL is the active process that leads to adaptation to a stressor and is defined as “achieving stability *via* change” (McEwen and Stellar, 1993). Previous findings indicated that higher allostatic load or allostatic overload was associated with poorer health outcomes in both general and clinical populations, such as poor physical health in re-employed populations with no good jobs (Chandola and Zhang, 2018), unexplained infertility in fertile women (Barrett et al., 2018), and overall mortality in patients with metastatic non-small cell lung cancer (Obeng-Gyasi et al., 2022), which supported the clinical efficacy of the trans-diagnostic identification of allostatic load and allostatic overload (Guidi et al., 2021). Thus, we believe that maintaining a low level of AL might be helpful for patients undergoing hemodialysis to decrease risks to health.

It has been found that lower serum Mg^2+^ was negatively correlated to AL levels in patients undergoing hemodialysis, which was the main novel finding in our study. Mg^2+^ loss in patients undergoing hemodialysis is less likely to attract the attention of clinicians, which has also been called a “neglected cation” (Alhosaini and Leehey, 2015). In fact, Mg^2+^ plays an important role in the homeostasis of organisms. It is not only a cofactor for several enzymes involved in most cellular processes (Jahnen-Dechent and Ketteler, 2012). Changes in the level of serum Mg^2+^ are also involved in a variety of pathological conditions (Tangvoraphonkchai and Davenport, 2018). Due to the controversy over the clinical significance of disturbances in magnesium homeostasis and the true risk of hypomagnesemia and hypermagnesemia, this “neglect” was proposed (Alhosaini and Leehey, 2015). A large study in Japan showed that there was a relationship between low magnesium concentrations and mortality, which means that hypomagnesemia was an important predictor of cardiovascular or non-cardiovascular mortality. Hypermagnesemia is also associated with higher mortality, but the intensity of correlation was smaller than that of hypomagnesemia (Sakaguchi et al., 2014).

Increasing risks for AL were connected to low serum Mg^2+^. Mg^2+^ was strongly linked to the cardiovascular system, inflammation, insulin resistance, and metabolic syndrome (Belin and He, 2007). Some studies found that magnesium deficiency is associated with an increased risk of hypertension (Liu and Dudley, 2020), diabetes (Piuri et al., 2021), and mortality (Sakaguchi et al., 2014). As mentioned previously, Mg^2+^ is an essential cofactor for many enzymes involved in glucose metabolism. In animal experiments, the onset of diabetes appears to be delayed in model rats supplemented with Mg^2+^, whereas animals with low Mg^2+^ levels show elevated serum glucose levels and altered lipid patterns (Mancuso et al., 2020). Other studies showed that magnesium supplementation improves the insulin resistance index and beta cell function and reduces hemoglobin A1c (HbA1c) levels in patients with type 2 diabetes mellitus (Rodrı´guez-Mora´n and Guerrero-Romero, 2003; Guerrero-Romero and Rodrı´guez-Mora´n, 2011). In our study, a strong negative correlation between Mg^2+^ and blood glucose was found, which is consistent with previous findings. In addition, Mg^2+^ has been found to be associated with dyslipidemia in the general population, and our study also indicated that Mg^2+^ was strongly correlated to related blood lipid indexes in the hemodialysis population. Our results also proved the evidence for the effect of Mg^2+^ on dyslipidemia, similar to a previous study (He et al., 2006). Moreover, it has been found that serum Mg^2+^ also had a strong correlation with diastolic blood pressure. However, no studies have focused on the connection between Mg^2+^ and blood pressure, but there are some studies reporting the effects of different magnesium concentrations in the dialyzate on blood pressure. For example, a study in Greece showed that hypotensive episodes during dialysis were least common in using the highest magnesium dialyzate and most common in using the lowest magnesium dialyzate. In addition, studies in Egypt and Iran also support this view (Kyriazis et al., 2004; Elsharkawy et al., 2006; Pakfetrat et al., 2010). However, there are also some opposite results. Different serum Mg^2+^ concentrations in the dialyzate have no significant effect on blood pressure in dialysis patients (Deng et al., 2015). Future studies are needed to verify whether a higher serum Mg^2+^ concentration has a protective effect on blood pressure control. Interestingly, we found that CRP was negatively correlated with Mg^2+^. Low Mg^2+^ status was accompanied by an elevation of CRP, and Mg^2+^ supplementation reduces CRP levels (Mahabir, 2014; Mazidi et al., 2018; Nielsen, 2018).

The present large-scale, multi-center study contributed to high reliability of the findings. However, some limitations should be addressed. First, a cross-sectional design could not provide causal associations, and prospective cohort studies are needed to verify causal associations between serum Mg^2+^ and AL in the future. Second, patients without complete biomarkers of AL were excluded, which may influence the results. To determine the impact of the excluded samples on the results, a sensitivity analysis was performed. The results revealed that there were some differences in age, family residence, hypertension, diabetes, cardiovascular disease, and antihypertensive and hypolipidemic medication. Consistent with the findings, participants included in this study may have overestimated the percentage of high AL. Therefore, the inclusion criteria which strictly controlled and stratified the analysis of patients with comorbidity are warranted. Third, the components of AL in different studies were not unified, which commonly consisted of the metabolic system, autonomic nervous system, and immune system. The neuroendocrine index of AL was lacking in the present study, but the indicators selected in our study were well-validated in the different biological systems, and the results were in line with those of our original hypothesis and previous studies. At last, it should also be mentioned that the generalizability of our findings to different ethnicities is unknown due to significant worldwide disparities in the clinical pattern and prognosis of hemodialysis patients.

## Conclusion

This is the first study to examine serum Mg^2+^ and AL in the hemodialysis population. Patients undergoing hemodialysis with lower levels of serum Mg^2+^ are more likely to be at higher health risks. In addition, this study also provides evidence for the effects of serum Mg^2+^ on blood lipids, glucose metabolism, blood pressure, and immunity. The findings could provide empirical evidence for the intervention of Mg^2+^ supplements on reducing the health risk of the hemodialysis population.

## Data Availability

The original contributions presented in the study are included in the article/Supplementary Material; further inquiries can be directed to the corresponding authors.

## Supplementary material

SUPPLEMENTARY TABLE S1
Sensitivity analysis of characteristics between the included sample and excluded sample.
