# Supplementary material for: A multi-center study on the association between serum magnesium levels and allostatic load in hemodialysis patients
Source: Front Physiol. 2022 Oct 3;13:963914. doi: 10.3389/fphys.2022.963914 (PMC9574054; doi:10.3389/fphys.2022.963914)
Supplement: Supplementary file 1 [file Table1.docx]

**Table S1** **Sensitivity analysis of characteristics between the included sample and**

**excluded sample.**

| Variables | M±SD/n (%) | Excluded sample  (n=1803) | Included sample  (n=1222) | *χ*^2^ / *t* value | *P* value |
| --- | --- | --- | --- | --- | --- |
| Age | 55.00±12.79 | 54.08±12.77 | 55.90±12.75 | -3.855 | <0.001 |
| Gender |  |  |  | 0.019 | 0.891 |
| Male | 1819(60.1) | 1086 (60.2) | 733(60.0) |  |  |
| Female | 1206(39.9) | 717 (39.8) | 489(40.0) |  |  |
| Family residence |  |  |  | 15.114 | <0.001 |
| Rural | 1352(44.7) | 858(47.6) | 494(40.4) |  |  |
| Urban | 1673(55.3) | 945(52.4) | 728(59.6) |  |  |
| Smoking | 462(15.3) | 278(15.4) | 184(15.1) | 0.074 | 0.786 |
| Drinking | 360(11.9) | 212(11.8) | 148(12.1) | 0.087 | 0.769 |
| Comorbidities |  |  |  |  |  |
| Diabetes | 732(24.2) | 396(22.0) | 336(27.5) | 12.154 | <0.001 |
| Hypertension | 2277(75.3) | 1288(71.4) | 989(80.9) | 34.781 | <0.001 |
| Cardiovascular disease | 426(14.1) | 232(12.9) | 194(15.9) | 5.202 | 0.023 |
| Medications |  |  |  |  |  |
| Antihypertensive | 2117(70.0) | 1317(73.0) | 800(70.4) | 19.554 | <0.001 |
| Hypolipidemic | 480(15.9) | 216(12.0) | 264(21.6) | 49.814 | <0.001 |
